# Supplementary material for: Filament organization of the bacterial actin MreB is dependent on the nucleotide state
Source: J Cell Biol. 2022 Apr 4;221(5):e202106092. doi: 10.1083/jcb.202106092 (PMC9195046; doi:10.1083/jcb.202106092)
Supplement: Table S1 — lists data collection and refinement statistics of S. citri MreB5 bound to ADP. [file JCB_202106092_TableS1.docx]

Table S1**. Data collection and refinement statistics of *S. citri* MreB5 bound to ADP**

| ScMreB5-ADP | |
| --- | --- |
| *Data Collection statistics* | |
| Collected at | Rigaku Micromax-007 |
| Wavelength (Å) | 1.5418 |
| Space Group | P1 |
| a, b, c (Å) | 37.9, 41.1, 56.3 |
| α, β, γ (˚) | 82.3, 74, 80.4 |
| Resolution (Å)* | 40.4-2.3 (2.38 – 2.3) |
| Number of unique reflections* | 12584 (1233) |
| R_merge_ (%)* | 0.12 (0.63) |
| R_pim_ (%)* | 0.11 (0.52) |
| CC_half_* | 0.98 (0.48) |
| Mean I/σI | 6.1 (1.5) |
| Completeness (%)* | 87.9 (86.2) |
| Redundancy* | 2.1 (2.1) |
| *Refinement statistics* | |
| Resolution (Å) | 36.14-2.3 |
| Number of unique reflections (test set) | 12580(657) |
| R_work_ / R_free_ (%) | 22.5/27.2 |
| Average B-factor (Å^2^) | 23.1 |
| Wilson B-factor (Å^2^) | 19.22 |
| *RMS deviations* | 0.002 |
| Bond lengths (Å) | 0.51 |
| Bond angles (˚) |  |
| *Ramachandran map statistics* |  |
| Favored (%) | 97.85 |
| Allowed (%) | 7 |
| Outliers (%) | 0 |

* Values in parenthesis denote the outer resolution shell
