# Supplementary material for: Filament organization of the bacterial actin MreB is dependent on the nucleotide state
Source: J Cell Biol. 2022 Apr 4;221(5):e202106092. doi: 10.1083/jcb.202106092 (PMC9195046; doi:10.1083/jcb.202106092)
Supplement: Table S2 — lists kobs values of WT and mutants. [file JCB_202106092_TableS2.docx]

Table S2. ***k_obs_* values of wildtype and mutants**

| **Protein** | **Activity [*k_obs_*(min^-1^)]** |
| --- | --- |
| ScMreB5^WT^ | 0.15 + 0.007 |
| ScMreB5^D12A^ | 0.02 + 0.008 |
| ScMreB5^D156A^ | 0.08 + 0.014 |
| ScMreB5^E134A^ | 0.01 + 0.004 |
| ScMreB5^T161A^ | 0.0005 + 0.001 |
| ScMreB5^D70A^ | 0.05 + 0.002 |
| ScMreB5^K57A^ | 0.10 + 0.01 |
| ScMreB5^WT^, N-terminal GFP | 0.12 + 0.01 |
