# Supplementary material for: Filament organization of the bacterial actin MreB is dependent on the nucleotide state
Source: J Cell Biol. 2022 Apr 4;221(5):e202106092. doi: 10.1083/jcb.202106092 (PMC9195046; doi:10.1083/jcb.202106092)
Supplement: Table S3 — lists primers and clones. [file JCB_202106092_TableS3.docx]

Table S3. **List of primers and clones**

| **Primer name** | **Sequence (‘5 → 3’)** | **Clones generated using the primers**  **(Vector-construct name)** |
| --- | --- | --- |
| ScM5-f | CTTTAAGAAGGAGATATACATATGAGACCAGAAACTAGACCATTTATTTC | pHis17-ScMreB5^WT^ |
| ScM5H6-r | GATGATGATGATGATGGGATCCTTTTCTTTTTTTACCTAATGTTGATAATAATCC |  |
| ScM5W-f | GAATGAAAATGATGAACATTTGGAAGAATGCTATTG |  |
| ScM5-k57a-f | CTATGATATGGTAGGAGCAACACACGGAGATATTAG | pHis17-ScMreB5^K57A^ |
| ScM5-D156-f | GGTCATTTAATCATTGCTATCGGTGGAGGAACAAC | pHis17-ScMreB5^D156A^ |
| ScM5-E134-f | GTTATCATTGAAGAAGCGGCTAAAATGGCCG | pHis17-ScMreB5^E134A^  pRep-ScMreB5^E134A^-NGFP |
| D12A-f | CCAGAAACTAGACCATTTATTTCTCTTGCGTTAGGAACTGCTAATG | pHis17-ScMreB5^D12A^ |
| ScM5-D70-f | GGTAACACCATTAGTAGCGGGAGTTATCGCAGACATGGAAGCTGCAC | pHis17-ScMreB5^D70A^ |
| ScM5-T161A-f | GGTGGAGGAGCGACTGATTTAGCTATTATTTCATCAGGTG | pHis17-ScMreB5^T161A^ |
| M5-I95A | CAAGAATGAAAATGATGAACGCGTGGAAGAATGCTATTGTATTATTAGC | pHis17-ScMreB5^I95A^ |
| M5-W96A | CAAGAATGAAAATGATGAACATTGCGAAGAATGCTATTGTATTATTAGC | pHis17-ScMreB5^W96A^ |
| M5-IWA | CAAGAATGAAAATGATGAACGCCGCGAAGAATGCTATTGTATTATTAGC | pHis17-ScMreB5^IWA^ |
| ScM5Ct10 del-r | GCTTTTAATGATGATGATGATGATGGGATCCTTTTTCTTGAAAATTATATAAACC | pHis17-ScMreB5^ΔC10^ |
| pREP_ScM5-f | GGCATGGATGAACTATACAAACATATGATGAGACCAGAAACTAGACCATTTATTTC | pRep-ScMreB5-NGFP |
| pREP_ScM5-r | GGCAAGGGAGACATTCCTTTTACCCGGGGATCCTTATTTTCTTTTTTTACCTAATGTTG |  |
