# Supplementary material for: Filament organization of the bacterial actin MreB is dependent on the nucleotide state
Source: J Cell Biol. 2022 Apr 4;221(5):e202106092. doi: 10.1083/jcb.202106092 (PMC9195046; doi:10.1083/jcb.202106092)
Supplement: SourceData F5 — contains original blots for Fig. 5. [file JCB_202106092_SourceDataF5.pdf]

SourceDataF5D

Anti-GFP

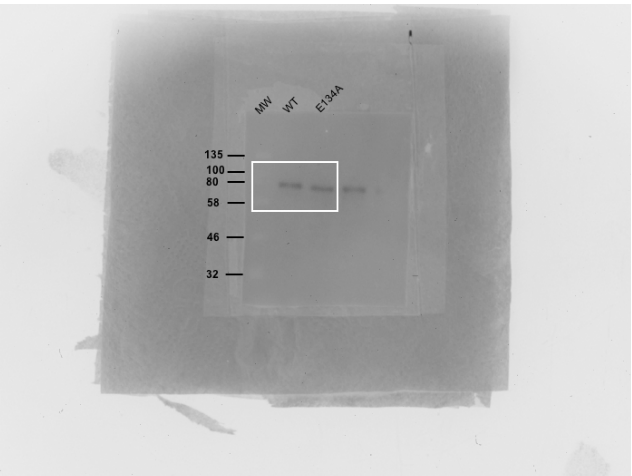

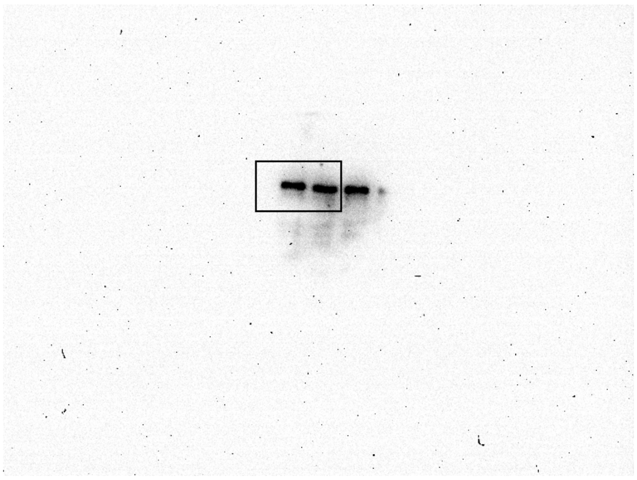

Anti-α-Tubulin

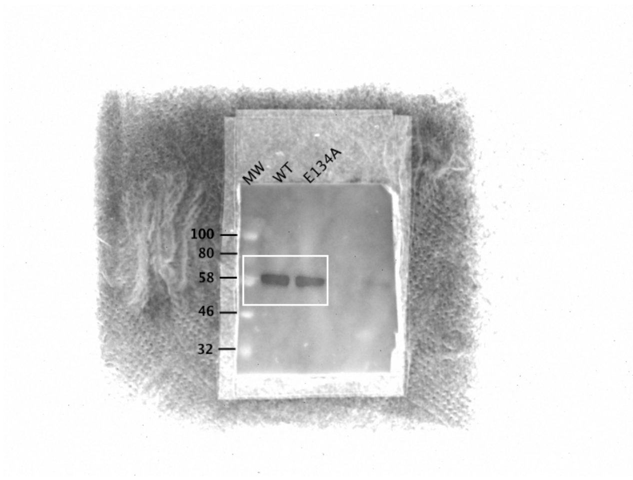

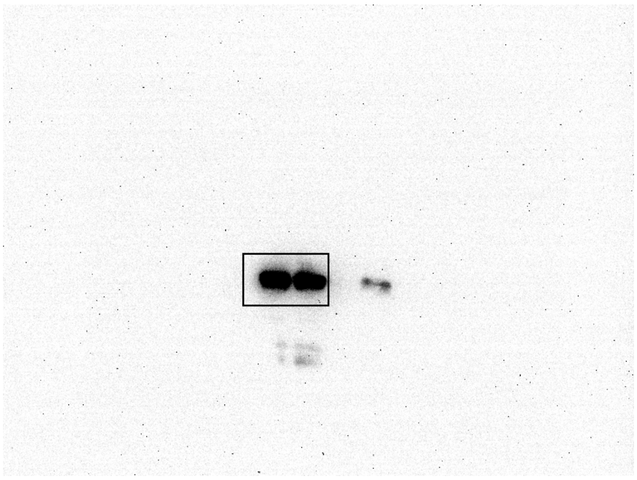

The data included in the figure was from the contrast-adjusted bottom panel (boxed portion only).
