# Supplementary figures and images for: Filament organization of the bacterial actin MreB is dependent on the nucleotide state
Source: J Cell Biol. 2022 Apr 4;221(5):e202106092. doi: 10.1083/jcb.202106092 (PMC9195046; doi:10.1083/jcb.202106092)

SourceDataF6E

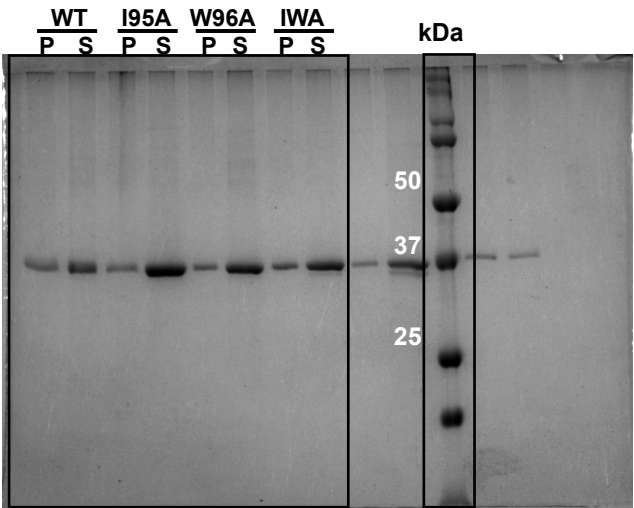

Supplement: SourceData F6 — contains original blots for Fig. 6. [file JCB_202106092_SourceDataF6.pdf]

SourceDataF7A

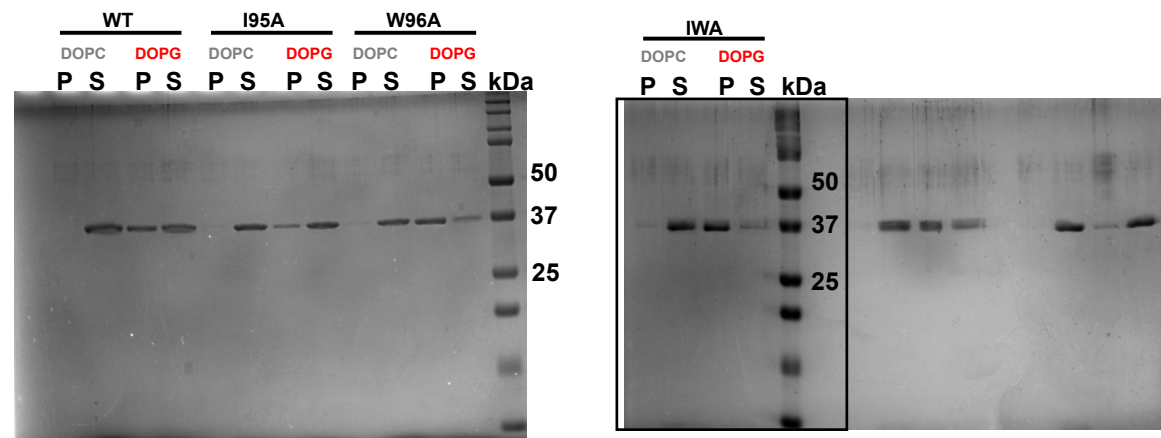

SourceDataF7D

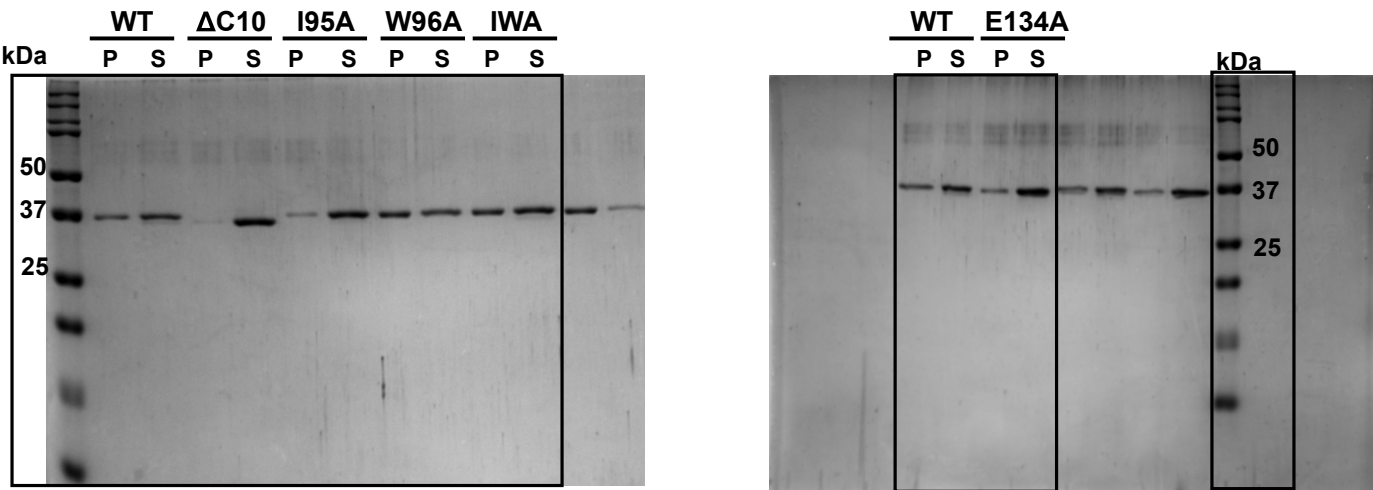

SourceDataF7G

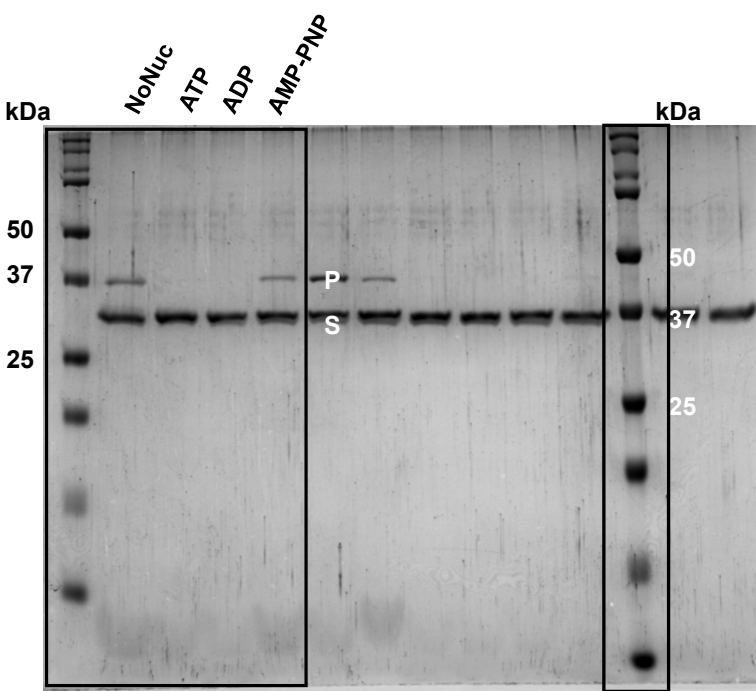

Supplement: SourceData F7 — contains original blots for Fig. 7. [file JCB_202106092_SourceDataF7.pdf]

# SourceDataFS3A

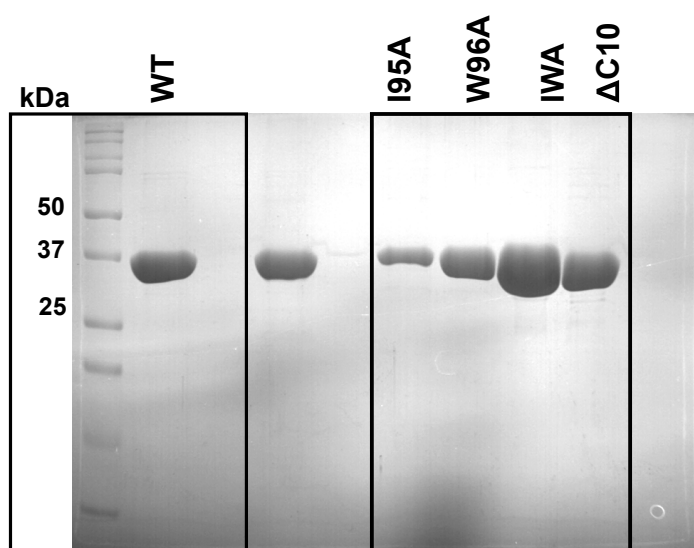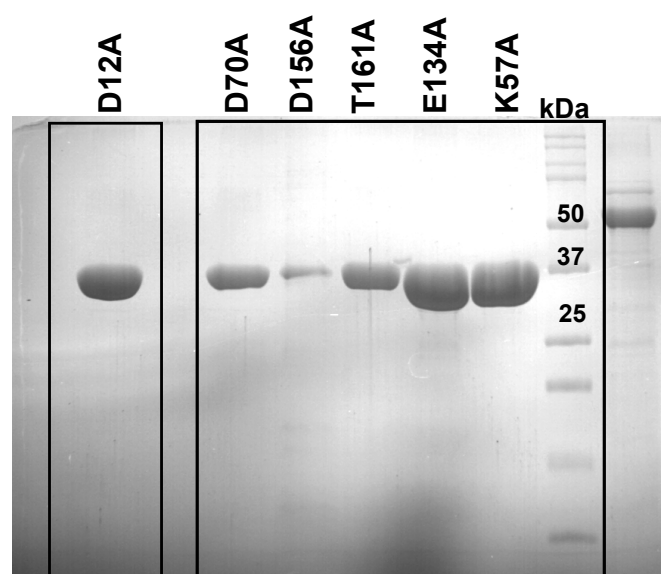

Supplement: SourceData FS3 — contains original blots for Fig. S3. [file JCB_202106092_SourceDataFS3.pdf]

SourceDataS4A

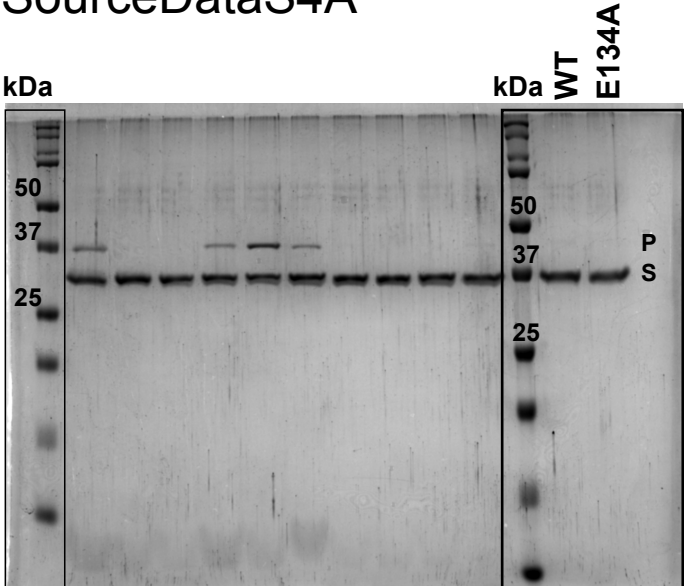

SourceDataS4B

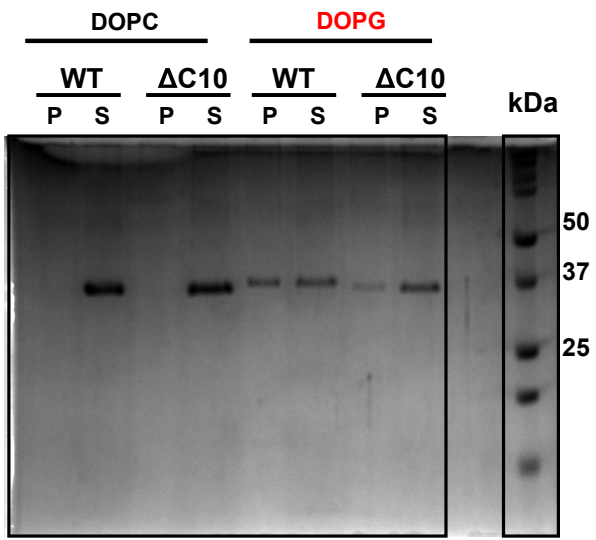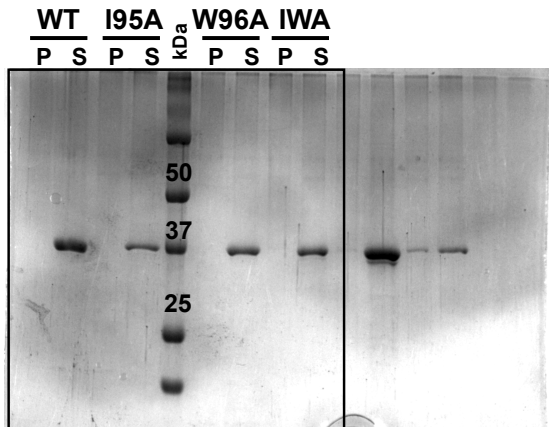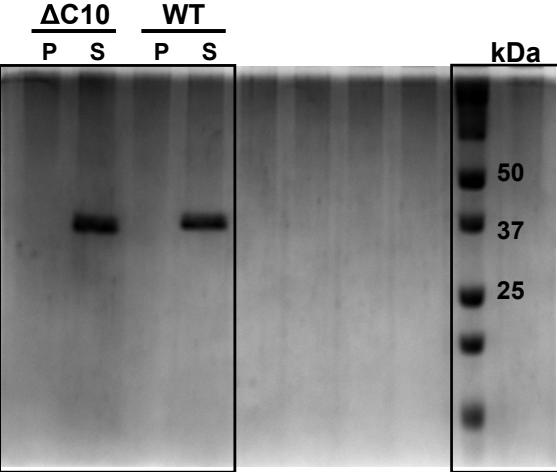

SourceDataS4D

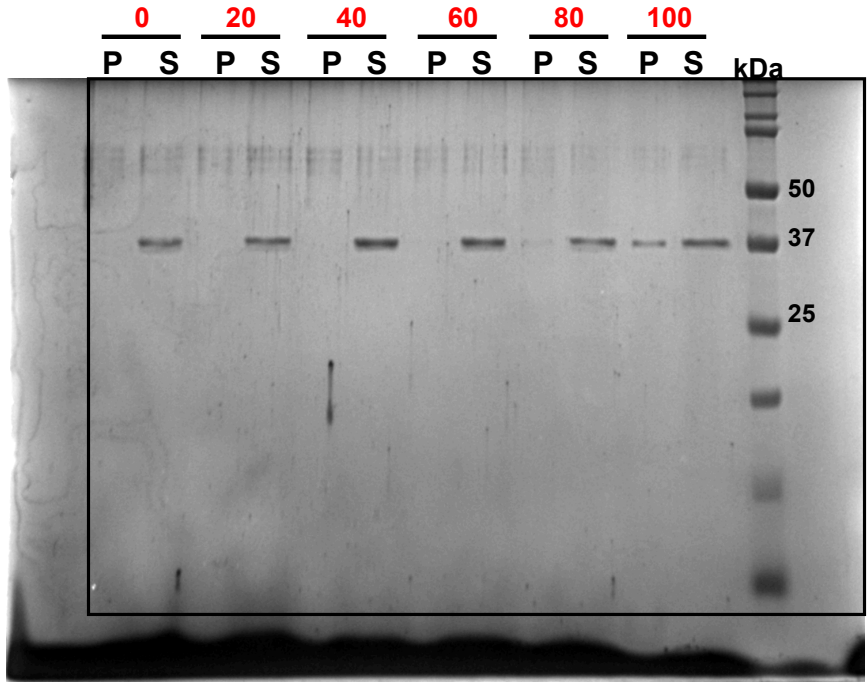

SourceDataS4E

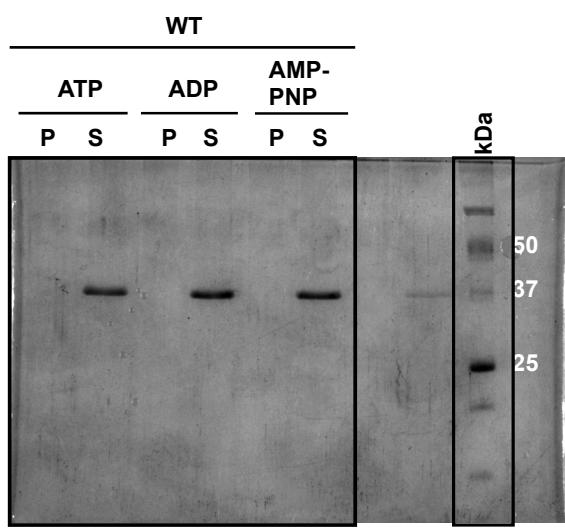

Supplement: SourceData FS4 — contains original blots for Fig. S4. [file JCB_202106092_SourceDataFS4.pdf]
